# A comparison of stem cell-related gene expression in the progenitor-rich limbal epithelium and the differentiating central corneal epithelium

**Published:** 2011-08-10

**Authors:** Teresa Nieto-Miguel, Margarita Calonge, Ana de la Mata, Marina López-Paniagua, Sara Galindo, María Fideliz de la Paz, Rosa M. Corrales

**Affiliations:** 1 Institute for Applied Ophthalmobiology (IOBA), University of Valladolid, Valladolid, Spain; 2 Networking Research Center on Bioengineering, Biomaterials and Nanomedicine (CIBER-BBN), Valladolid, Spain; 3 Institut Universitari Barraquer, Universitat Autonoma de Barcelona, Barcelona, Spain

## Abstract

**Purpose:**

Corneal epithelium is maintained by a population of stem cells (SCs) that have not been identified by specific molecular markers. The objective of this study was to find new putative markers for these SCs and to identify associated molecular pathways.

**Methods:**

Real time PCR (rt-PCR) was performed in 24 human limbal and central corneal epithelial samples to evaluate the gene expression profile of known corneal epithelial SC-associated markers. A pool of those samples was further analyzed by a rt-PCR array (RT^2^-PCR-A) for 84 genes related to the identification, growth, maintenance, and differentiation of SCs.

**Results:**

Cells from the corneal epithelium SC niche showed significant expression of ATP-binding cassette sub-family G member 2 (*ABCG2*) and cytokeratin (*KRT*)*15*,  *KRT14*, and *KRT5* genes. RT^2^-PCR-A results indicated an increased or decreased expression in 21 and 24 genes, respectively, in cells from the corneal SC niche compared to cells from the central corneal epithelium. Functional analysis by proprietary software found 4 different associated pathways and a novel network with the highest upregulated genes in the corneal SC niche. This led to the identification of specific molecules,  chemokine (C-X-C motif) ligand 12 (CXCL12), islet-1 transcription factor LIM/homeodomain (ISL1), collagen-type II alpha 1 (COL2A), neural cell adhesion molecule 1 (NCAM1), aggrecan (ACAN), forkhead box A2 (FOXA2), Gap junction protein beta 1/connexin 32 (GJB1/Cnx32), and Msh homeobox 1 (MSX1), that could be used to recognize putative corneal epithelial SCs grown in culture and intended for transplantation. Other molecules, NCAM1 and GJB1/Cnx32,  potentially could be used to positively purify them, and Par-6 partitioning defective 6 homolog alpha (PARD6A) to negatively purify them.

**Conclusions:**

Knowledge of these gene and molecular pathways has provided a better understanding of the signaling molecular pathways associated with progenitor-rich limbal epithelium. This knowledge potentially could give support to the design and development of innovative therapies with the potential to reverse corneal blindness arising from ocular surface failure.

## Introduction

The cornea is the clear front of the eye through which light enters on its way to the retina. The corneal outer surface is covered by a stratified squamous nonkeratinized epithelium that resists constant attrition caused by exposure-induced dryness and potential light-induced damage [1]. To cope with this demand, constant renewal and maintenance of the corneal epithelium is achieved by stem cells (SCs) located at the circular border of the cornea in a region known as the corneoscleral limbus. The basal epithelial cells of the limbal region are not homogeneous, but rather consist of diverse populations of SCs, transient amplifying cells, and terminally differentiated cells for which the total number and distribution are unknown [1-4]. Limbal SC deficiency (LSCD) syndrome occurs if limbal epithelial SCs (LESCs) are critically reduced and/or dysfunctional due to a multitude of conditions including genetic disorders (i.e., anirida), cicatrizing-autoimmune pathologies (i.e., Steven-Johnson syndrome, mucous membrane pemphygoid), severe infections, or external factors such as chemical or thermal burns, ultraviolet and ionizing radiation, contact lens wear, and multiple surgeries. The consequence of LSCD is a chronic pain inflammatory syndrome and loss of vision, greatly affecting quality of life and productivity [5].

Current treatment of LSCD relies on the inhibition of inflammation, protection, and provision of LESCs for reconstruction of the damaged corneas [5-7]. Strategies based on transplantation of ex vivo expanded LESCs are becoming widely accepted today. The most frequently chosen technique includes harvesting autologous or allogenic limbal tissue that is then cultivated on amniotic membranes or fibrin matrices. Transplantation of these cultured cells has shown promising results [8-12]. However, it is usually not known what percentage of the transplanted cells is actually composed of SCs. It is likely that the success of each transplantation depends upon the number of SCs included. For example, enrichment of transplants with LESCs expressing the marker p63 increases the success rate [10]. It is therefore essential to improve the purity of the LESCs being transplanted to ensure good long-term transplantation results.

Identifying LESCs is crucial for enrichment and characterization. Unfortunately, to date, no direct methods have been established because no single specific LESC marker is known. A variety of SC markers has been proposed to identify the LESC population. In addition, a diversity of differentiation markers has also been proposed to differentiate LESCs from terminally differentiated corneal epithelial cells [13-16]. Until now, the combination of positive and negative SC markers seems to be the most trustworthy way to characterize the putative SCs in the limbal epithelium. Typically, the major positive markers used are the transcription factor p63, the drug-resistance transporter ATP-binding cassette sub-family G member 2 (ABCG2), and some cytokeratins (KRTs) like KRT15 and KRT14. Among the most used as negative markers are KRT3 and KRT12, and the gap junction protein connexin 43, which are all typical of terminally differentiated cells [10,13,15,16].

Recently, great efforts have been made toward the identification of new molecular markers that may better distinguish LESCs from transient amplifying cells and terminally differentiated cells [16,17]. However, the variety of putative LESC markers and their role for the identification the LESC population is controversial [15,18]. The finding of new molecules that specifically identify LESCs would significantly enhance the purity of LESCs grown in culture and intended for transplantation. In addition, a better understanding of the molecular signaling pathways associated with the stemness of the limbal epithelium could facilitate a better diagnosis of LSCD and could also give support to the design and development of new and promising treatments. Therefore to discover new putative LESC markers, we analyzed the expression of 84 genes related to the identification, growth, maintenance, and differentiation of human SCs. Using a real time reverse transcription polymerase chain reaction array (RT^2^-PCR-A) with human corneal and limbal samples, we found increased and decreased expression of selected genes operating in 4 different pathways constituting signaling networks in the cells from the limbal stem cell niche.

## Methods

### Epithelial cell collection

Human tissue was used in accordance with the Declaration of Helsinki. Normal human corneoscleral tissues (n=24) were obtained 3 to 5 days post-mortem from the Barraquer Eye Bank (Barcelona, Spain). Limbal and central cornea epithelial cells were obtained using a modification of a previously described method [19-23]. In brief, a 7.5 mm trephine was used to isolate the cornea from the limbus, and the epithelium in the central button of the cornea was scraped to harvest differentiating epithelial cells for analysis of gene expression. Later, each corneoscleral rim was trimmed, and the endothelial layer and iris remnants were removed. The limbal rim was incubated with dispase II (5 mg/ml; STEMCELL Technologies, Grenoble, France) at 37 °C for 2 h. The limbal epithelial sheets were then collected and treated with 0.25% trypsin with 0.03% EDTA (Invitrogen-Gibco, Inchinnan, UK) at 37 °C for 10 min to isolate single cells. There were, therefore, 24 samples of 2 different types of epithelial cells: differentiating corneal epithelial cells and stem cell-containing population of limbal epithelial cells derived from the corneal epithelial stem cell niche.

### RNA isolation and reverse transcription

Total RNA was extracted by Qiagen RNeasy Mini Kit (QIAGEN Inc., Valencia, CA) under standard conditions, and treated with RNase-free DNase following our previously described method [24-26]. Briefly, samples were collected in RNA lysis buffer (1:100 β-mercaptoethanol-buffer RLT), purified in QIAshredder columns, and treated with RNase-Free DNase I Set (QIAGEN Inc.) following the manufacturer’s instructions. Agarose gel electrophoresis and ethidium bromide staining were used to check the integrity and size distribution of the purified RNA. The first strand of cDNA was synthesized with random hexamer using M-MuLV Reverse Transcriptase (Amersham Pharmacia Biotech Europe GmbH, Barcelona, Spain) [24-26].

### Real time polymerase chain reaction (rt-PCR)

The cDNA from the limbal and corneal epithelial cells was mixed with Taqman assay primers and minor groove binder probes specific for glyceraldehyde 3-phosphate dehydrogenase (*GAPDH*), *KRT3*, *KRT5*, *KRT7*, *KRT12*, *KRT14*, *KRT15*, *KRT19*, *p63* and *ABCG2* (Table 1) and with a Taqman Universal PCR Master Mix AmpErase UNG (Applied Biosystems, Foster City, CA) in a 7500 Real Time PCR System (Applied Biosystems) according to the previously described method [27-31]. An aliquot of 2 μl containing 20 ng of cDNA was used for PCR in a total volume of 20 μl containing: 7 µl double-distilled water, 1 μl of 20× target primers and probe, 10 µl of 2× Taqman Universal PCR Master Mix. PCR parameters consisted of uracil N-glycosylane activation at 50 °C for 2 min, pre-denaturation at 95 °C for 2 min, followed by 40 cycles of denaturation at 95 °C for 15 s, and annealing and extension at 60 °C for 1 min.

**Table 1:** Oligonucleotide primers and probes used for real time PCR

**Gene name**	**Gene symbol**	Assay ID*
Glyceraldehyde-3-phosphate dehydrogenase	*GAPDH*	4352934E
Protein p63	*P63*	Hs00978338_m1
ATP-binding cassette, sub-family G, member 2	*ABCG2*	Hs00184979_m1
Keratin 3	*KRT3*	Hs00365080_m1
Keratin 5	*KRT5*	Hs00361185_m1
Keratin 7	*KRT7*	Hs00818825_m1
Keratin 12	*KRT12*	Hs00165015_m1
Keratin 14	*KRT14*	Hs00559328_m1
Keratin 15	*KRT15*	Hs00267035_m1
Keratin 19	*KRT19*	Hs01051611_gh

*Identification number from Applied Biosystems (www.appliedbiosystems.com).

Assays were performed in triplicate. A nontemplate control and total RNA without retrotranscription were included in all experiments to evaluate PCR and DNA contamination of the reagents. *GAPDH* was used as an endogenous reference for each reaction to correct for differences in the amount of total RNA added. To verify the validity of using *GAPDH* as an internal standard control, the efficiencies of the genes and *GAPDH* amplifications were compared.

The comparative cycle threshold (Ct) method, where the target fold=2^-ΔΔCt^, was used for analyzing the results (Applied Biosystems User Bulletin, No.2, P/N 4303859) [27-31]. Corneal mRNA served as the calibrator control. The results were reported as a fold upregulation when the fold-change for limbal cells was greater than one compared to corneal cells. If the fold-change was less than one, the negative inverse of the result was reported as a fold down-regulation. Significant differences (p<0.05) were evaluated by Student’s *t*-test.

### Real time PCR array

The samples were pooled, creating 4 groups of 6 each, and used for further study. Analysis using a real time PCR (rt-PCR) array was performed according to the manufacturer’s recommendations using the Human Stem Cell RT^2^ Profiler™ (SuperArray Bioscience, Izasa, S.A., Barcelona, Spain) that used SYBR® Green I dye detection.

We studied the expression of 5 housekeeping genes, 3 RNAs and PCR quality controls, and 84 human genes related to:

1. SC specific markers (cell cycle regulators, chromosome and chromatin modulators, genes regulating symmetric/asymmetric cell division, self-renewal, cytokines and growth factors, genes regulating cell-cell communication, cell adhesion molecules and metabolism),
2. SC differentiation markers (embryonic, hematopoietic, mesenchymal, and neural cell lineage markers), and
3. Signaling pathways important for SC maintenance (Notch and Wnt pathways).

The following components were mixed in a 5-ml tube: 1,275 μl of the 2× SuperArray PCR Master Mix, 102 μl (100 ng) of the diluted first strand cDNA synthesis reaction, and 1,173 μl double-distilled H_2_O. This mixture and template cocktail (25 μl each) was added to each well of the PCR array. Real time PCR (7500 Real Time PCR System) was then performed as follows: 10 min at 95 °C, 40 cycles of 15 s at 95 °C, and 1 min at 60 °C. Assays were performed in duplicate. A melting curve program was run and a dissociation curve was generated for each well in the entire plate to verify the identity of each gene amplification product.

For data analysis, the Ct method was performed using an Excel-based PCR Array Data Analysis template that was downloaded from the SuperArray website. This program automatically performed the following calculations and interpretation of gene expression based upon threshold cycle data from a real-time instrument:

1. Changed to 35 all Ct values greater than 35 and Ct values not detected. At this point, any Ct value equaled to 35 was considered a negative call.
2. Examined the threshold cycle values of the genomic DNA control, reverse transcription control, and positive PCR control wells.
3. Calculated the ΔCt for each gene in each plate.

We used the average of the five housekeeping gene Ct values as a normalization factor. The results are reported as a fold upregulation or down-regulation in the same way as previously explained for real time PCR (above).

### Pathway analysis

Excel spreadsheets containing gene identifier lists together with the corresponding expression values were uploaded into Ingenuity Pathways Analysis (IPA; Ingenuity® Systems, Redwood City, CA) to identify relationships among the genes of interest. The basis of the IPA program consisted of the Ingenuity Pathways Knowledge Base (IPKB) that was derived from known functions and interactions of genes published in the literature. Thus, the IPA tool allowed the identification of biologic networks, global functions, and functional pathway(s) of a particular data set. Each gene identifier was mapped to its corresponding gene object in the IPKB. Networks of the genes were then algorithmically generated based on their connectivity.

Each gene product was assigned to functional and sub-functional categories. IPA software then used the associated library of canonical pathways to identify the most significant ones in the data set. Benjamini-Hochberg multiple testing correction was used to calculate a p-value to determine the probability that each biologic function or canonical pathway assigned to the data set was due to chance alone. In addition, significance of the association between the data set and the canonical pathway was calculated as a ratio of the number of genes from the data set that mapped to the pathway divided by the total number of genes that map to the canonical pathway. The ‘Pathway Designer’ tool of the IPA software was used for the graphical representation of the molecular relationships between gene products. Gene products were represented as nodes, and the biologic relationship between two nodes was represented as an edge (line). All edges were supported by at least one reference from the literature, from a textbook, or from canonical information stored in the IPKB.

## Results

### Real time PCR analysis for corneal and limbal epithelial cell markers

**Figure 1:**
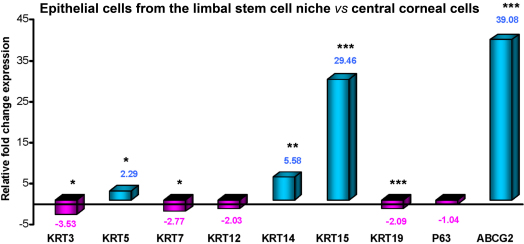
Relative expression of cytokeratins (*KRTs*), *p63,* and *ABCG2* genes. Real time PCR was performed in 24 samples of limbal and corneal epithelial cells. Mean mRNA expression in limbal epithelial cells was expressed relative to that corneal epithelial cells. Positive values indicated relatively greater expression in limbal cells, while negative values indicated relatively less expression in limbal. Significance was analyzed by Student’s *t*-test. *p<0.05, **p<0.001, ***p<0.00001.

To select the purest population of corneal and limbal epithelial cells, we performed rt-PCR assays to evaluate the expression of markers considered to be abundant in the limbal stem cell niche. These markers included *KRT14*, *KRT15*, *ABCG2*, and transcription factor *p63* [13,20,32-35]. For terminally differentiated corneal epithelial cells, we looked for the expression of *KRT3*, *KRT7*, and *KRT12* [3,36], as well as for other cytokeratins like *KRT5* and *KRT19* [15]. In the 24 samples analyzed, all of the studied *KRT* genes were expressed (Figure 1). In the limbal epithelial cells, expression was significantly reduced for most cytokeratin genes that are normally expressed in large amounts in terminally differentiated epithelial cells [15,35]. The reductions for *KRT3*, *KRT7*, *KRT12*, and *KRT19*, which varied between 2.03 and 3.54 fold, were all significant except for *KRT12* (p<0.05 for *KRT3* and *KRT17*, p>0.05 for *KRT12*, and p<0.00001 for *KRT19,* Figure 1). In contrast, *KRT5*, *KRT14*, and *KRT15* were more highly expressed in the limbal than the corneal epithelial samples, with increases ranging from 2.29 to 29.46 fold (p<0.05, <0.001, and <0.00001, respectively, Figure 1).

Gene expression of associated LESC niche markers *ABCG2* and *p63* were found in all of the samples analyzed. Expression levels of *ABCG2* were 39.1 fold greater in the limbal epithelial cell population than in the corneal epithelial one (p<0.00001, Figure 1). However, expression of transcription factor p63 was the same in both cell populations.

Summarizing our results so far, the purest SC-containing population of limbal epithelial cells had significantly higher expression of *ABCG2* (39 fold), *KRT15* (29.5 fold), *KRT14* (5.6 fold), and *KRT5* (2.3 fold) than did the corneal epithelial cell population. Furthermore, the limbal cells had significantly lower expression of *KRT3*, *KRT7*, and *KRT19*. Neither *KRT12* nor *p63* were useful as gene markers to differentiate between the two cell populations.

### Real time PCR array

The 24 samples previously analyzed by real time PCR were pooled to perform the PCR array. The dissociation curve was analyzed for the 84 genes studied, and no DNA contamination was detected. The results indicated increased expression of 21 genes and decreased expression of 24 genes for limbal cells compared to corneal cells. Eleven genes had a greater than ninefold increased expression and 10 genes had a greater than fourfold decreased expression (Table 2).

**Table 2:** Up- and down-regulated genes in cells of the limbal epithelial stem cell niche.

**Symbol**	**Entrez gene name**	Location*	**Type**	**Fold change**
*CXCL12*	Chemokine (C-X-C motif) ligand 12 (stromal cell-derived factor 1)	Extracellular space	Cytokine	+26.45
*ISL1*	ISL LIM homeobox 1	Nucleus	Transcription regulator	+20.90
*COL2A1*	Collagen, type II, alpha 1	Extracellular space	Other	+19.47
*NCAM1*	Neural cell adhesion molecule 1	Plasma membrane	Other	+11.82
*ABCGG2*	ATP-binding cassette, sub-family G (WHITE), member 2	Plasma membrane	Transporter	+10.25
*KRT15*	Keratin 15	Cytoplasm	Other	+10.00
*ACAN*	Aggrecan	Extracellular space	Other	+9.24
*FOXA2*	Forkhead box A2	Nucleus	Transcription regulator	+9.24
*GJB1*	Gap junction protein, beta 1, 32 kDa	Plasma membrane	Transporter	+9.24
*MSX1*	Msh homeobox 1	Nucleus	Transcription regulator	+9.24
*CD8B*	CD8b molecule	Plasma membrane	Other	+9.17
*DHH*	Desert hedgehog homolog (Drosophila)	Extracellular space	Peptidase	−17.66
*CDC2*	Cell division cycle 2, G1 to S and G2 to M	Nucleus	Kinase	−9.33
*GJA1*	Gap junction protein, alpha 1, 43 kDa	Plasma membrane	Transporter	−6.89
*CCNA2*	Cyclin A2	Nucleus	Other	−6.71
*PARD6A*	Par-6 partitioning defective 6 homolog alpha (C. elegans)	Plasma membrane	Other	−5.13
*KAT2A*	K(lysine) acetyltransferase 2A	Cytoplasm	Enzyme	−5.07
*DVL1*	Dishevelled, dsh homolog 1 (Drosophila)	Cytoplasm	Other	−4.61
*JAG1*	Jagged 1 (Alagille syndrome)	Extracellular space	Growth factor	−4.54
*S100B*	S100 calcium binding protein B	Cytoplasm	Other	−4.34
*FRAT1*	Frequently rearranged in advanced T-cell lymphomas	Cytoplasm	Other	−4.28

Genes with higher (+) and lesser (-) expression in limbal epithelial cells compared to terminally differentiated corneal epithelial cells. Fold change was calculated by PCR array using the comparative Ct method. *Indicates cellular location where protein is expressed.

Among the 11 most upregulated genes (Table 2) from the limbal SC niche, three coded for extracellular space proteins (chemokine [C-X-C motif] ligand 12 [*CXCL12*], collagen-type II alpha 1 [*COL2A*], and aggrecan [*ACAN*]), three for transcription factors located at the nucleus (islet-1 transcription factor LIM/homeodomain [*ISL1*], forkhead box A2 [*FOXA2*], and Msh homeobox 1 [*MSX1*]), four for plasma membrane proteins (neural cell adhesion molecule 1 [*NCAM1*], *ABCG2*, Gap junction protein beta 1 [*GJB1*], and CD8b molecule [*CD8B*]), and only one for a cytoplasmic protein (*KRT15*). Among them, the most upregulated expression was for the chemokine *CXCL12* gene with 26.45 fold increased expression.

Among the 10 most down-regulated genes (Table 2), two coded for extracellular space proteins (Desert hedgehog homolog [*DHH*]**and Jagged 1 [*JAG1*]), two for nuclear proteins (Cell division cycle 2 [*CDC2*]**and Cyclin A2 [*CDCNA2*]), two for plasma membrane proteins (Gap junction protein alpha 1 [*GJA1*] and Par-6 partitioning defective 6 homolog alpha [*PARD6A*]), and four for cytoplasmic proteins (K[lysine] acetyltransferase 2A [*KAT2A*], Dishevelled dsh homolog 1 [*DVL1*], S100 calcium binding protein B [*S100B*], and Frequently rearranged in advanced T-cell lymphomas [*FRAT1*]). Among them, the most down-regulated expression was for the *DHH *peptidase gene with 17.66 fold decreased expression.

#### Signaling pathways

**Figure 2:**
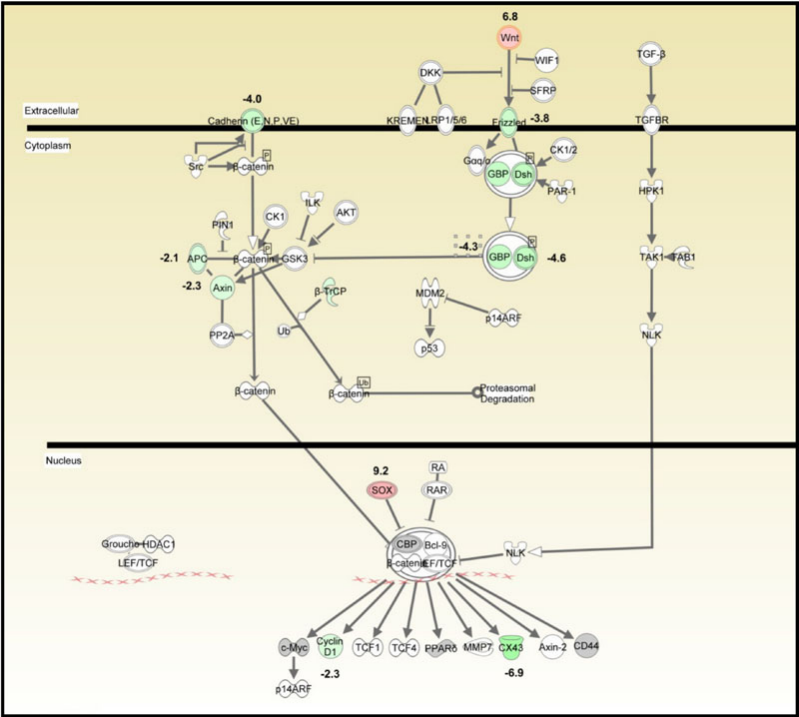
Wnt/β-catenin signaling pathway generated by Ingenuity Pathway Analysis (IPA). The IPA depicted the genes involved, their interactions, and the cellular and metabolic reactions that constituted the pathway. Colored molecules represented genes that appeared in the data set studied. Red and green molecules were up- and down-regulated, respectively, in limbal epithelial cells. Gray molecules did not meet the user defined cutoff of 2.

Seventy canonical signaling pathways were significantly affected across the entire data set identified by IPA (Table 3, Figure 2). The highest upregulated gene was *SOX* (9.2 fold, Figure 2) in the Wnt/β-catenin signaling pathway, also known as *SRY* (sex determining region Y)-box 2, Entrez Gene 6736). The most down-regulated gene was *GJA1* (6.9 fold), also known as gap junction protein, alpha 1 (Entrez Gene 2697).

**Table 3:** Ingenuity canonical pathways that were most significantly affected.

**Ingenuity Canonical Pathways**	-Log(B-H P-value)*	**Ratio**	**Molecules**
Wnt/β^2^-catenin Signaling	8.54E+00	6.67E-02	SOX2, CDH2, GJA1, AXIN1, FRAT1, DVL1, BTRC, FZD1, CCND1, WNT1, APC
Notch Signaling	3.28E+00	9.76E-02	NOTCH2, DLL1, DTX1, JAG1
Cell Cycle: G1/S Checkpoint Regulation	2.58E+00	6.78E-02	CCNE1, HDAC2, BTRC, CCND1
Actin Cytoskeleton Signaling	1.49E+00	2.20E-02	FGF4, CDC42, ACTC1, APC, FGF1
Aryl Hydrocarbon Receptor Signaling	1.34E+00	2.58E-02	CCNA2, CCNE1, ALDH1A1, CCND1
Clathrin-mediated Endocytosis	1.34E+00	2.42E-02	FGF4, CDC42, ACTC1, FGF1
Axonal Guidance Signaling	1.34E+00	1.52E-02	CDC42, BMP2, FZD1, WNT1, BMP1
FGF Signaling	1.27E+00	3.49E-02	FGF4, FGFR1, FGF1
Ephrin Receptor Signaling	1.27E+00	2.07E-02	CDC42, AXIN1, CXCL12, FGF1
T Cell Receptor Signaling	1.11E+00	2.78E-02	CD8A, CD3D, CD8B
Cell Cycle: G2/M DNA Damage Checkpoint Regulation	1.07E+00	4.65E-02	BTRC, CDC2
Tight Junction Signaling	8.86E-01	1.83E-02	CDC42, ACTC1, PARD6A
NF-Κ°B Signaling	8.86E-01	2.08E-02	HDAC2, BMP2, BTRC
Calcium-induced T Lymphocyte Apoptosis	8.21E-01	3.28E-02	HDAC2, CD3D
Leukocyte Extravasation Signaling	7.08E-01	1.55E-02	CDC42, CXCL12, ACTC1
BMP signaling pathway	7.08E-01	2.50E-02	BMP2, BMP1
Regulation of Actin-based Motility by Rho	7.08E-01	2.17E-02	CDC42, ACTC1
PTEN Signaling	6.12E-01	2.02E-02	CDC42, CCND1
Fcγ^3^ Receptor-mediated Phagocytosis in Macrophages and Monocytes	5.94E-01	1.92E-02	CDC42, ACTC1
CD28 Signaling in T Helper Cells	4.92E-01	1.65E-02	CDC42, CD3D
Cytotoxic T Lymphocyte-mediated Apoptosis of Target Cells	4.92E-01	3.85E-02	CD3D
Glucocorticoid Receptor Signaling	4.92E-01	1.09E-02	HSPA9, BGLAP, CD3D
Hepatic Fibrosis / Hepatic Stellate Cell Activation	4.73E-01	1.48E-02	FGFR1, FGF1
Sonic Hedgehog Signaling	4.34E-01	3.23E-02	CDC2
Ascorbate and Aldarate Metabolism	4.12E-01	1.22E-02	ALDH1A1
Calcium Signaling	3.39E-01	9.71E-03	HDAC2, ACTC1
Retinol Metabolism	3.39E-01	1.56E-02	ALDH1A1
Integrin Signaling	3.39E-01	1.01E-02	CDC42, ACTC1
Huntington’s Disease Signaling	3.39E-01	8.62E-03	HDAC2, HSPA9
Histidine Metabolism	3.39E-01	9.01E-03	ALDH1A1
GM-CSF Signaling	3.39E-01	1.54E-02	CCND1
Activation of IRF by Cytosolic Pattern Recognition Receptors	3.39E-01	1.37E-02	ADAR
Macropinocytosis	3.39E-01	1.43E-02	CDC42
CCR5 Signaling in Macrophages	3.39E-01	1.16E-02	CD3D
Neurotrophin/TRK Signaling	3.39E-01	1.32E-02	CDC42
Caveolar-mediated Endocytosis	3.39E-01	1.25E-02	ACTC1
PXR/RXR Activation	3.39E-01	1.16E-02	ALDH1A1
LPS-stimulated MAPK Signaling	3.39E-01	1.27E-02	CDC42
Bile Acid Biosynthesis	3.39E-01	1.03E-02	ALDH1A1
Chemokine Signaling	3.39E-01	1.30E-02	CXCL12
L^2^-alanine Metabolism	3.39E-01	1.01E-02	ALDH1A1
VDR/RXR Activation	3.39E-01	1.25E-02	BGLAP
Butanoate Metabolism	3.39E-01	7.75E-03	ALDH1A1
Pyruvate Metabolism	3.39E-01	6.90E-03	ALDH1A1
CTLA4 Signaling in Cytotoxic T Lymphocytes	3.39E-01	1.16E-02	CD3D
TGF-β^2^ Signaling	3.39E-01	1.16E-02	BMP2
Lysine Degradation	3.39E-01	6.94E-03	ALDH1A1
Propanoate Metabolism	3.39E-01	7.94E-03	ALDH1A1
Apoptosis Signaling	3.39E-01	1.06E-02	CDC2
p53 Signaling	3.39E-01	1.15E-02	CCND1
VEGF Signaling	3.39E-01	1.05E-02	ACTC1
SAPK/JNK Signaling	3.39E-01	1.08E-02	CDC42
Glycerolipid Metabolism	3.39E-01	6.90E-03	ALDH1A1
Valine, Leucine and Isoleucine Degradation	3.39E-01	9.35E-03	ALDH1A1
FXR/RXR Activation	3.39E-01	1.00E-02	FOXA2
Glycolysis/Gluconeogenesis	3.33E-01	7.09E-03	ALDH1A1
Nicotinate and Nicotinamide Metabolism	3.33E-01	7.75E-03	CDC2
fMLP Signaling in Neutrophils	3.03E-01	8.00E-03	CDC42
Arginine and Proline Metabolism	3.01E-01	5.62E-03	ALDH1A1
PI3K/AKT Signaling	3.00E-01	7.41E-03	CCND1
Protein Ubiquitination Pathway	2.60E-01	4.95E-03	BTRC
Inositol Phosphate Metabolism	2.60E-01	5.78E-03	CDC2
B Cell Receptor Signaling	2.54E-01	6.54E-03	CDC42
CXCR4 Signaling	2.51E-01	6.10E-03	CXCL12
IL-8 Signaling	2.51E-01	5.46E-03	CCND1
Tryptophan Metabolism	2.51E-01	4.20E-03	ALDH1A1
RAR Activation	2.51E-01	5.49E-03	ALDH1A1
Role of NFAT in Regulation of the Immune Response	2.51E-01	5.38E-03	CD3D
Fatty Acid Metabolism	2.51E-01	5.29E-03	ALDH1A1
NRF2-mediated Oxidative Stress Response	2.51E-01	5.46E-03	ACTC1
LPS/IL-1 Mediated Inhibition of RXR Function	2.29E-01	5.05E-03	ALDH1A1

*The p-value was calculated using the Benjamini-Hochberg (BH) method. The ratio was calculated as the number of genes in a given pathway that met the cutoff criteria, two in this case, divided by the total number of genes that made up the pathway.

#### Predicted functional effects

The IPA program determined if groups of genes with significantly changed expression levels were associated with altered biologic functions and diseases (Table 4). Here IPA identified 71 functional categories that were significantly affected. The most prominent cellular and molecular functions implicated were cellular development, cell death, gene expression, cellular assembly and organization, and cellular growth and proliferation. The most frequent significant physiologic system developments were tissue, organismal, embryonic, nervous system, and organ development.

**Table 4:** Molecules significantly associated with relevant functions and diseases.

**Category**	**B-H P-value**	**Molecules**
Cellular Development	3.49E-13–1.53E-02	37
Tissue Development	3.61E-10–1.53E-02	31
Organismal Development	3.91E-09–4.75E-03	22
Embryonic Development	2.37E-08–1.53E-02	26
Cell Death	3.61E-08–1.53E-02	35
Gene Expression	2.67E-07–1.53E-02	32
Cellular Assembly and Organization	2.67E-07–1.53E-02	22
Nervous System Development and Function	2.67E-07–1.53E-02	29
Cancer	3.1E-07–1.53E-02	40
Cellular Growth and Proliferation	5.45E-07–1.53E-02	39
Cell Morphology	5.45E-07–1.53E-02	23
Cell Cycle	1.15E-06–1.53E-02	17
Organ Development	1.69E-06–1.36E-02	20
Skeletal and Muscular Disorders	3.01E-06–1.53E-02	11
Renal and Urological Disease	4.17E-06–1.36E-02	7
Genetic Disorder	5.66E-06–1.53E-02	39
Developmental Disorder	7.55E-06–1.53E-02	17
Connective Tissue Development and Function	4.09E-05–1.53E-02	21
Skeletal and Muscular System Development and Function	4.09E-05–1.5E-02	17
Hematological System Development and Function	8.41E-05–1.53E-02	18
Hematopoiesis	8.41E-05–1.53E-02	13
Neurologic Disease	1.35E-04–1.53E-02	27
Lymphoid Tissue Structure and Development	1.86E-04–1.53E-02	9
Auditory and Vestibular System Development and Function	2.51E-04–8.77E-03	5
Cell-To-Cell Signaling and Interaction	2.92E-04–1.53E-02	23
Cellular Movement	2.98E-04–1.53E-02	22
Cellular Function and Maintenance	3.24E-04–1.53E-02	14
Cardiovascular System Development and Function	3.82E-04–1.53E-02	14
Hepatic System Disease	3.82E-04–1.53E-02	14
Reproductive System Disease	5.45E-04–1.53E-02	19
Gastrointestinal Disease	5.48E-04–1.53E-02	21
Tissue Morphology	6.35E-04–1.53E-02	18
Energy Production	6.56E-04–6.56E-04	3
Molecular Transport	6.56E-04–1.53E-02	3
Nucleic Acid Metabolism	6.56E-04–1.53E-02	4
Small Molecule Biochemistry	6.56E-04–1.53E-02	9
Organ Morphology	1.04E-03–1.53E-02	13
Tumor Morphology	1.13E-03–1.53E-02	10
Metabolic Disease	1.33E-03–1.33E-03	3
DNA Replication. Recombination. and Repair	1.5E-03–1.53E-02	11
Connective Tissue Disorders	1.54E-03–7.79E-03	10
Humoral Immune Response	2.21E-03–3.6E-03	4
Visual System Development and Function	2.21E-03–7.79E-03	4
Psychological Disorders	2.25E-03–2.25E-03	6
Infection Mechanism	2.47E-03–9.23E-03	4
Post-Translational Modification	2.69E-03–1.53E-02	11
Carbohydrate Metabolism	2.81E-03–2.81E-03	5
Lipid Metabolism	2.81E-03–2.81E-03	3
Drug Metabolism	2.81E-03–1.53E-02	3
Endocrine System Development and Function	2.81E-03–1.53E-02	4
Hair and Skin Development and Function	3.04E-03–1.53E-02	6
Reproductive System Development and Function	3.45E-03–1.53E-02	4
Hematological Disease	5.19E-03–1.53E-02	8
Cardiovascular Disease	5.28E-03–5.28E-03	4
Cell-mediated Immune Response	6.31E-03–1.3E-02	5
Organismal Injury and Abnormalities	6.88E-03–6.88E-03	3
Digestive System Development and Function	7.14E-03–7.14E-03	3
Organismal Survival	7.53E-03–7.53E-03	12
Hepatic System Development and Function	8.76E-03–8.76E-03	2
Respiratory System Development and Function	8.76E-03–1.53E-02	3
Antigen Presentation	1.23E-02–1.23E-02	2
Inflammatory Disease	1.53E-02–1.53E-02	2
Cell Signaling	1.53E-02–1.53E-02	2
Protein Trafficking	1.53E-02–1.53E-02	2
Vitamin and Mineral Metabolism	1.53E-02–1.53E-02	2
Renal and Urological System Development and Function	1.53E-02–1.53E-02	2
Auditory Disease	1.53E-02–1.53E-02	1
Cellular Compromise	1.53E-02–1.53E-02	1
Dermatological Diseases and Conditions	1.53E-02–1.53E-02	1
Immune Cell Trafficking	1.53E-02–1.53E-02	1
RNA Post-Transcriptional Modification	1.53E-02–1.53E-02	1

*The p-value was calculated using the Benjamini-Hochberg (BH) method.

#### Gene networks

**Figure 3:**
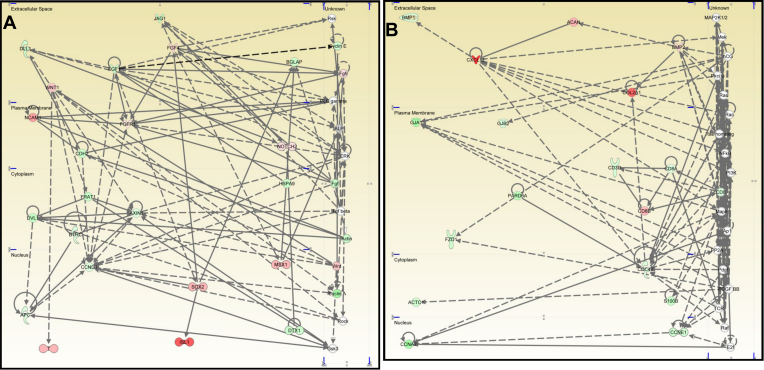
Networks generated by IPA related to the development and the function of the auditory, vestibular, skeletal and muscular systems and to the cancer development. Auditory and vestibular system development and function, organ development, and cancer network (**A**), and cancer, connective tissue development and function, skeletal and muscular system development and function network (**B**) generated by IPA. The networks contained nodes composed of genes/gene products and edges that indicated a relationship between the nodes in the cellular and subcellular locations indicated. Classes of nodes were indicated by shape to represent different functionalities. Colored molecules represented genes that appeared in the data set studied. Red and green molecules were upregulated and down-regulated, respectively, in the limbal epithelial cells. Gray molecules did not meet the user defined cutoff of 2. White indicated the molecule was added from the IPKB.

The IPA program constructed 4 gene networks that were significantly interconnected. The first network (Figure 3A) contained 22 genes concerned with the auditory and vestibular system development and function, organ development, and cancer. Upregulated genes included *FGF4*, *FGFR1*, *ISL1*, *MSX1*, *NCAM1*, *NOTCH2*, *SOX2*, T, and *WNT1*. Down-regulated genes included *APC*, *AXIN1*, *BGLAP*, *BTRC*, *CCND1*, *CDH2*, *DLL1*, *DTX1*, *DVL1*, *FGF1*, *FRAT1*, *HSPA9*, and *JAG1*.

The second network (Figure 3B) contained 17 genes associated with cancer, connective tissue development and function, and skeletal and muscular system development and function. Upregulated genes included *ACAN*, *BMP2*, *CD8B*, *COL2A1*, and *CXCL12*. Down-regulated genes included *ACTC1*, *BMP1*, *CCNA2*, *CCNE1*, *CD3D*, *CD8A*, *CDC42*, *FZD1*, *GJA1*, *GJB2*, *PARD6A*, and *S100B*.

**Figure 4:**
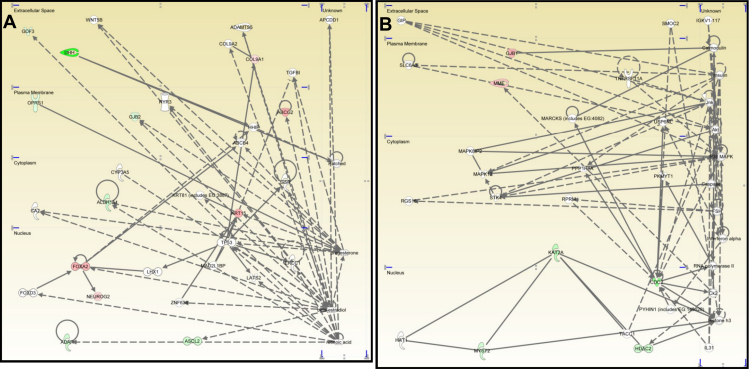
Networks generated by IPA related to drug metabolism, small molecule biochemistry and cell morphology and to cancer, cell cycle, skeletal and muscular disorders. Drug metabolism, small molecule biochemistry and cell morphology network (**A**), and cancer, cell cycle, skeletal and muscular disorders network (**B**) generated by IPA. The network contained nodes (gene/gene product) and edges (indicating a relationship between the nodes) showing the cellular/subcellular location as indicated. Function classes of nodes were indicated by shape to represent functional class. Colored molecules represented genes that appeared in the data set studied. Red and green molecules were upregulated and down-regulated, respectively, in limbal epithelial cells. Gray molecules did not meet the user defined cutoff of 2. White indicated the molecule was added from the IPKB.

*ALDH1* occupied a central position in the third network (Figure 4A), which contained 12 genes concerned with drug metabolism, small molecule biochemistry, and cell morphology expression. Upregulated genes included *ABCG2*, *COL9A1*, *FOXA2*, *KRT15*, and *NEUROG2*. Down-regulated genes included *ADAR*, *ALDH1A1*, *ASCL2*, *COL9A2DHH*, *GDF3*, *GJB2*, and *OPRS1*.

Finally, the fourth network (Figure 4B) contained 6 genes affecting cancer, cell cycle, and skeletal and muscular disorders. There were 2 upregulated genes, *GJB1* and *MME*, and 4 down-regulated, *CDC2*, *HDAC2*, *KAT2A*, and *MYST2*.

#### Customized gene network

**Figure 5:**
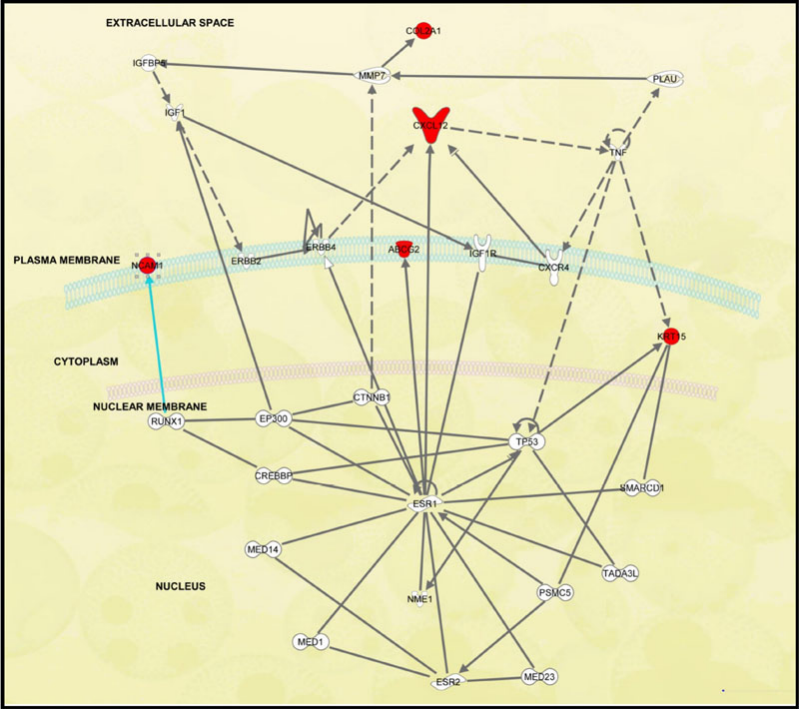
Customized gene network based upon the six most highly upregulated limbal epithelial cell genes. We explored possible functional relationships between the six highest upregulated limbal epithelial cells genes (in red) using the IPKB. Our customized pathway contained nodes composed of genes/gene products and edges that indicated a relationship between the nodes in the cellular and subcellular locations indicated. White indicates that the molecule was added from the IPKB.

Using the IPKB, we explored possible functional relationships among the six highest upregulated limbal epithelium progenitor-rich cell genes (Table 2): (1) *CXCL12*, (2) *ISL1*, (3) *COL2A1*, (4) *NCAM1*, (5) *ABCG2*, and (6) *KRT15*. We obtained a network with 29 genes. The protein products of 14 genes were active in the nucleus, one in the cytoplasm, six in the plasma membrane, and seven in the extracellular space (Figure 5).

*CXCL12*, also called stromal cell-derived factor 1 (*SDF1*), encodes for small cytokines that belong to the intercrine family (Entrez Gene 6387). We chose it as the central gene in the network because in humans it directly or indirectly interacts with the other genes that we added. Among the 6 most upregulated genes, only *ISL1*, which encodes for a member of the LIM/homeodomain family of transcription factors and may play an important role in regulating insulin gene expression (Entrez Gene 3670), did not have any connections with other genes in this network.

## Discussion

Isolation and characterization of tissue specific SCs to study their functional properties is one of the main research aspirations for regenerative medicine. In the context of ocular surface therapy, the ability to identify, purify, and characterize LESCs is an essential goal. However, the lack of LESC specific markers has been an obstacle for their isolation and subsequent biologic and functional characterization. Using cells isolated from the limbal SC niche, we compared the expression profile of 84 SC phenotype-related genes with cells from the differentiating central corneal epithelium zone. Our goal was to provide new information for molecules that are predominantly expressed in the stem cell-containing population of human limbal epithelial cells. Knowledge regarding these LESC potential markers could be used to enhance isolation of the cells and develop a better understanding of their biologic functions.

To know the gene expression pattern of the isolated cell samples, we first performed a PCR analysis for corneal and limbal epithelial markers. The limbal epithelial cell population expressed high levels of *ABCG2*, *KRT5*, *KRT14* and *KRT15* and low levels of *KRT3*, *KRT7*, and *KRT19*. Unexpectedly, we did not find significant differences between limbal and corneal epithelial cells for transcription factor *p63* expression. In 2001, Pellegrini et al. [33] proposed p63 as the first positive marker of LESCs. This has generated a certain level of controversy because several groups have since found that p63 is also expressed by most of the terminally differentiated basal epithelial cells throughout the cornea [18,37,38]. Our findings are consistent with the idea that p63 is not specific enough to be a definitive marker for LESCs, although perhaps it could be helpful for identifying incompletely differentiated corneal epithelial cells [18]. It is worth noting that the α isoform of ΔNp63 has been proposed to be a rather more specific and useful marker for LESCs than the other isoforms of this transcription factor [10,39].

Several microarray studies have attempted to identify markers and signaling pathways associated with different ocular surface cell phenotypes [32,40-49]. We chose the RT^2^-PCR-A system because it utilizes real-time PCR in combination with microarray analysis to detect the simultaneous expression of many genes. We used IPA to analyze our results from the PCR array, creating three different analysis types that responded to three different questions: (1) What well characterized cell signaling and metabolic canonical pathways are most relevant to our data set? (2) What regulatory networks exist among the genes and proteins of our data set? (3) What previously unknown, unique customized networks that can serve as biologic models are present in our data set?

Among the 84 genes we studied, 11 were highly upregulated and 10 were highly down-regulated; however less highly regulated genes may also be important in relation to SC properties. The most highly expressed in the limbal epithelium progenitor-rich cells compared to central corneal epithelial cells was the chemokine *CXCL2*. To explore molecular signatures of progenitor cells, we further analyzed six highly expressed genes, starting with the chemokine *CXCL12*, to create our customized gene network with a total of 29 molecules.

Chemokines are 8- to 10-kDa proteins that are potent activators and chemoattractants for different leukocyte subpopulations and some non-hematopoietic cells such as epithelial cells, fibroblasts, and endothelial cells [50]. CXCL12 and its receptor CXCR4 are expressed in cultured human corneal fibroblasts [51]. They may play a key role in angiogenesis and be involved in ocular neovascularization as well as in the recruitment of inflammatory or vascular endothelial cells to sites of corneal injury. In a recent microarray analysis of pig limbal side population cells, CXCR4 had the greatest overexpression ratio [42]. CXCR4 is also upregulated in pig and human conjunctiva side population cells [41,42]. Based on all of these findings, the CXCL12/CXCR4 pair could serve as a suitable marker to identify ocular surface SCs in a species-independent way. CXCL12/CXCR4 signaling is also critical for the mobilization and recruitment of mesenchymal SCs (MSCs) to infarcted hearts and fracture sites in bones [52,53]. Additionally, Ye et al. [54] recently reported that systemically transplanted bone marrow MSCs can engraft to injured cornea and promote wound healing by differentiation, proliferation, and synergizing with hematopoietic SCs. Thus we hypothesize that corneal homing of MSCs after ocular surface wounding could be mediated by release of CXCL12 from limbal epithelial cells and corneal fibroblast. Potentially, CXCL12 topical administration could be used to enhance MSC homing to injured corneal and limbal areas, facilitating the regenerative processes.

In addition to locating the SCs of the epithelium, the ideal SC marker should also allow for isolation and enrichment of viable SCs from a heterogeneous epithelial cell population. For that reason, cell surface proteins such as cell-cell and cell-matrix adhesion molecules, as well as cell surface receptors, may be the best candidates for new positive and negative putative LESC markers. Based on our results and others [15,20], the plasma membrane transporter ABCG2 appears to be the most useful cell surface marker for the identification and isolation of LESCs.

An example of a negative potential marker, one that indicates the absence of SC properties, could be *PARD6A*. This gene is a member of the PAR6 family and encodes a cell membrane protein involved in the control of epithelial cell polarity and tight junction assembly [55,56] and in epithelial-to-mesenchymal transition [57]. Expression of *PARD6A* in cells from the limbal stem cell niche was reduced fivefold compared to the corneal epithelial cells.

Another such negative marker is the gap junction protein connexin 43 (*GJA1*) that is abundantly expressed in the corneal but not in the limbal epithelium [19,58,59]. Membrane channel connexins (Cxs) form gap junctions that have been implicated in the homeostatic regulation of multicellular systems [60]. It is assumed that SCs of the limbal epithelium lack connexins and metabolite transfer capacity due to apparent self-sufficiency and absence of necessity for direct cell-to-cell communication [58]. However, our results showed upregulated expression of a related gene, Cx32 (*GJB1*), in limbal cells which was reported to be absent in human corneal epithelial cells [46]. Furthermore, Figueira et al. [32] recently described the expression of Cx32 in human fetal limbus and in cultured adult primary limbal explant epithelium. Similarly, hematopoietic cells were assumed not to express Cxs; however, hematopoietic SCs express Cx32 in response to chemical insult and also while maintaining the quiescent, noncycling state of primitive hematopoietic progenitor cells [61,62]. Although further investigations are required to confirm the role of Cx32 in LESCs, we propose this cellular surface protein as a new putative positive marker for the identification and isolation of human LESCs.

Expression of the neural cell adhesion molecule 1 (NCAM1) was highly upregulated in the limbal epithelial cells. NCAM is broadly expressed during development and plays a essential role in cell division, migration, and differentiation [63]. A decrease in NCAM expression during the development of the ocular lens has been associated with lens epithelial cell differentiation [64]. However NCAM is also expressed in cells of many fully developed tissues and organs including the cornea and lens epithelium [65]. For that reason, we believe it is not specific enough to serve as a potential single LESC marker.

The limbal epithelium may contain a higher proportion of immune-related cells such as macrophages, lymphocytes, and antigen presenting cells than does the central corneal epithelium [66,67]. Thus the presence of significant portions of marker transcripts derived from these kinds of cells is not surprising. The best example of this is CD8, a plasma membrane specific marker of T cells [68], that was overexpressed in the limbal-derived cells. This confirms the greater presence of immune-related cells in the limbal epithelium than in the corneal epithelium [66,67].

Analysis of our RT^2^-PCR-A data with IPA software recognized that the most significantly affected canonical pathway was Wnt/β2-catenin signaling, consistent with the recent findings of Bian et al. [43]. Wnt signaling is involved in practically every aspect of embryonic development and also controls homeostatic self-renewal in several adult tissues [69]. Among the studied molecules that belong to this pathway, *SOX2* and *Wnt* were the highest upregulated genes, 9.2 and 6.8 fold, respectively. The *SOX2* gene encodes a member of the SRY-related HMG-box (SOX) family of transcription factors implicated in the regulation of embryonic development and in the determination of cell fate [70]. Wnt signaling is required for the establishment of hair follicles, playing a key role in the activation of bulge SCs to progress toward hair formation [69,71]. Zhou et al. [48] prepared a transcriptional profile of mouse limbal and corneal epithelial basal cells. Consistent with our results, they found elevated expression of certain genes that were also upregulated in the hair follicular bulge SCs, suggesting the existence of a common cluster of epithelial SC genes. As we found, they also detected an elevated expression of the *Sry* gene in mouse limbal basal cells, associating it with increased proliferation. They proposed that it is involved in SC activation, maintaining proliferative capacity needed for expansion of precursor cell populations, and for wound healing [48]. Similarly, Figueira et al. [32] in a microarray analysis to identify phenotypic markers of human limbal SCs in fetal and adult corneas, detected that *Wnt-4* was differentially overexpressed in fetal limbus compared with central cornea. Its expression was restricted to the basal and immediate parabasal limbal epithelium of both the adult and fetal corneas. They suggested that, since Wnt-4 functions in diverse developmental phases involved in common morphogenic events, it was not surprising that this gene was expressed by the basal limbal epithelium that plays a crucial role in differentiation [32]. Wnt-4 overexpression, together with high levels of KRT15, KRT14, and P-cadherin in limbal basal epithelium cells, was in concordance with the molecular expression profile of stratified epithelial tissues. These data are in complete agreement with our RT^2-^PCR-A results that confirm an upregulated expression for both *Wnt* and *KRT15* molecules in limbal-derived epithelial cells.

Analysis of our RT^2^-PCR-A data with IPA software also constructed 4 networks that are distinct from canonical pathways because they were generated de novo based on our input data. The resulting networks require further studies to find the most useful genes for defining a potential LESC profile.

### 

#### Conclusions

In conclusion, our study has led to the identification of novel molecules, CXCL12, ISL1, COL2A, NCAM1, ACAN, FOXA2, GJB1/Cnx32, and MSX1, that potentially could serve to recognize LESCs. Other markers, NCAM1 and GJB1/Cnx32 positively and PARD6A negatively, could be used to separate the stem cell-containing population of limbal epithelial cells derived from limbal niche cells grown in culture and intended for transplantation. Furthermore, the functional analysis of our results has provided a better understanding of the signaling molecular pathways associated with the progenitor-rich limbal epithelium. This knowledge potentially could give support to the design and development of innovative therapies with the potential to reverse corneal blindness arising from ocular surface failure due to LSCD.
